# Expression and Prognosis Analysis of SUMOylation Regulators in Oral Squamous Cell Carcinoma Based on High-Throughput Sequencing

**DOI:** 10.3389/fgene.2021.671392

**Published:** 2021-06-29

**Authors:** Yutong Meng, Xiaozhi Li

**Affiliations:** ^1^Department of Stomatology, Shengjing Hospital of China Medical University, Shenyang, China; ^2^Department of Neurosurgery, Shengjing Hospital of China Medical University, Shenyang, China

**Keywords:** oral squamous cell carcinoma, SUMOylation, post-translational modification, prognosis, biomarkers

## Abstract

**Introduction:**

Oral squamous cell carcinoma (OSCC) originates from oral mucosal epithelial cells, accounting for more than 90% of oral cancers. The relationship between the expression and prognostic role of SUMOylation regulators in OSCC is rarely studied.

**Materials and methods:**

The expression and survival data of OSCC were derived from TCGA and GEO databases. Wilcoxon test was used to determine the differential expression of the SUMOylation regulators. A prognostic model based on SUMOylation regulator-related genes was constructed by Cox regression. Gene set enrichment analysis was applied to predict the potential biological functions that the genes might be involved in.

**Results:**

RANBP2 and SENP6 had the highest SNV frequency. Eleven genes including PIAS3, RANBP2, USPL1, SENP6, SENP2, SENP5, SAE1, UBA2, PIAS4, UBE2I, and SENP3 were highly expressed in OSCC. The prognostic model based on nine SUMOylation-regulated genes (TRIM37, UFM1, FUBP1, CCNT1, FXR1, HMG20A, RANBP3, SPATA5, and DDX23) had a strong ability to predict the prognosis of OSCC.

**Conclusion:**

This study might provide targets for prognostic evaluation and targeted therapy of patients with OSCC.

## Introduction

Oral squamous cell carcinoma (OSCC) originates from oral mucosal epithelial cells, accounting for more than 90% of oral cancers (Roy et al., 2019). Its 5-year survival rate is only about 56 –60% (Neville and Day, 2002; Petersen, 2003). Tobacco, alcohol, and poor oral hygiene are the main risk factors for OSCC (Moore et al., 2000; Joseph and D’Souza, 2012). The treatment of OSCC includes surgery, radiotherapy, and chemotherapy (Chinn and Myers, 2015). Based on the research of the molecular mechanism and the development of targeted drugs, precision medicine provides a new method to rescue tumor progression (Harsha et al., 2020).

SUMOylation is a post-translational modification that can reversibly connect SUMO (small ubiquitin-like modifier) to lysine residues in the substrates (Celen and Sahin, 2020; Kroonen and Vertegaal, 2020). The process of SUMOylation is catalyzed by the cascade reaction of E1 activating enzyme (SAE1 and UBA2), E2 conjugating enzyme (UBE2I), and E3 ligase (PIAS1, PIAS2, PIAS3, PIAS4, and RANBP2). Moreover, deSUMOylation is the opposite process of SUMOylation, which releases SUMO molecules. This process is catalyzed by SUMO proteases (SENP1, SENP2, SENP3, SENP5, SENP6, SENP7, and USPL1). SUMOylation is involved in cell localization, protein activity regulation, and protein stability changes, which regulates cell division, signal transfer, and cell metabolism (Dhingra and Zhao, 2019; Chang and Yeh, 2020; Roy and Sadanandom, 2021). It has been reported that the expression of SENP5 is elevated in OSCC and is related to the degree of tumor differentiation (Ding et al., 2008). However, the relationship between the expression and prognostic role of SUMOylation regulators in OSCC is rarely studied.

This study analyzed the expression of SUMOylation regulators in OSCC based on high-throughput sequencing and then constructed a prognostic model based on the SUMOylation regulator-related genes. This work might provide directions for the study of the mechanism of SUMOylation in OSCC.

## Materials and Methods

### Data Acquisition

The expression and survival data of OSCC were derived from TCGA and GEO databases (TCGA:^1^; GEO:^2^, GSE41613). Cases with overall survival time or total follow-up time less than 30 days and incomplete survival data were excluded. Finally, 323 cases of OSCC in TCGA, 32 cases of normal tissue, and 96 cases of OSCC in GEO were selected for follow-up analysis. In addition, single nucleotide variant (SNV) data were obtained from the TCGA database.

### SNV and Expression of SUMOylation Regulators in OSCC

Single nucleotide variant data of OSCC were analyzed by “maftools” package of R language. After extracting the expression of the SUMOylation regulators from the TCGA database, Wilcoxon test was used to determine the differential expression of the SUMOylation regulators. In addition, we analyzed the expression differences of SUMOylation regulators among different stages and grades.

### The Prognostic Model of OSCC

The Pearson correlation coefficient > 0.6 was used as selection criteria to screen the SUMOylation regulator-related genes. By using R language “caret” package, the TCGA dataset of OSCC was divided into a training group and the internal validation group at a ratio of 7:3. Meanwhile, the GEO dataset was used as the external validation group. In the TCGA training group, prognostic genes of OSCC were assessed by univariate Cox regression. Furthermore, we constructed the prognostic model by multivariate Cox regression. The risk score of every sample was calculated accordingly. Kaplan–Meier survival curve was applied to compare overall survival difference between high-risk and low-risk groups. ROC curve was introduced to evaluate the specificity and sensitivity of the model.

### Gene Set Enrichment Analysis

In this study, “clusterProfiler” package of R language was applied to perform GO and KEGG enrichment analyses on SUMOylation regulator-related genes to predict the potential biological functions that might be involved in.

### Statistical Analysis

The analyses and drawing of this work were all based on R language (4.0.2). When *P* was less than 0.05, the results were considered to be statistically different.

## Results

### SNV and Differential Expression of SUMOylation Regulators

**FIGURE 1:**
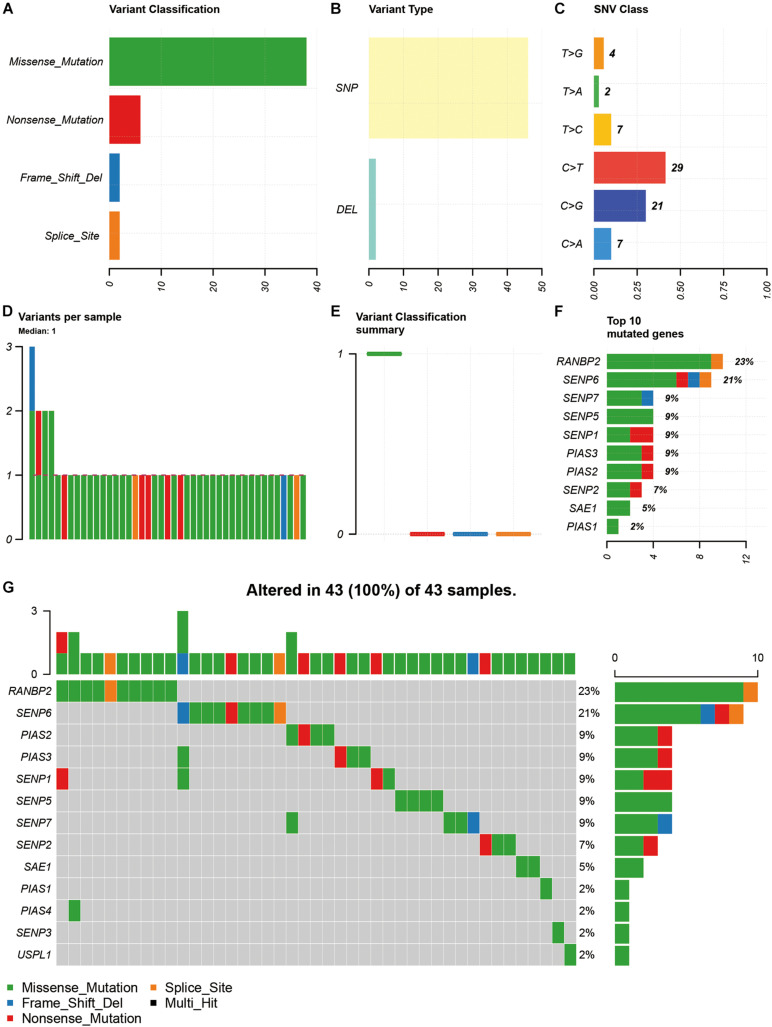
Summary of SNV of SUMOylation regulators in OSCC. **(A–C)** The SNV classification and type of OSCC. **(D,E)** SNV in specific samples. **(F)** The frequencies of SNV of SUMOylation regulators. **(G)** Waterfall plot of SNV in the TCGA samples.

Among the 15 SUMOylation regulators, missense mutation, SNP, and C > T were the most common types of SNV (Figures 1A–C). In addition, the majority of samples (93.02%) had only one SNV of SUMOylation regulators (Figures 1D,E). RANBP2 and SENP6 had the highest SNV frequency, accounting for 23 and 21% of all mutation cases, respectively. Other SUMOylation regulators with SNV included SENP7, SENP5, SENP1, PIAS3, PIAS2, SENP2, SAE1, and PIAS1 (Figure 1F). The distribution of SNV in OSCC samples was shown in Figure 1G.

**FIGURE 2:**
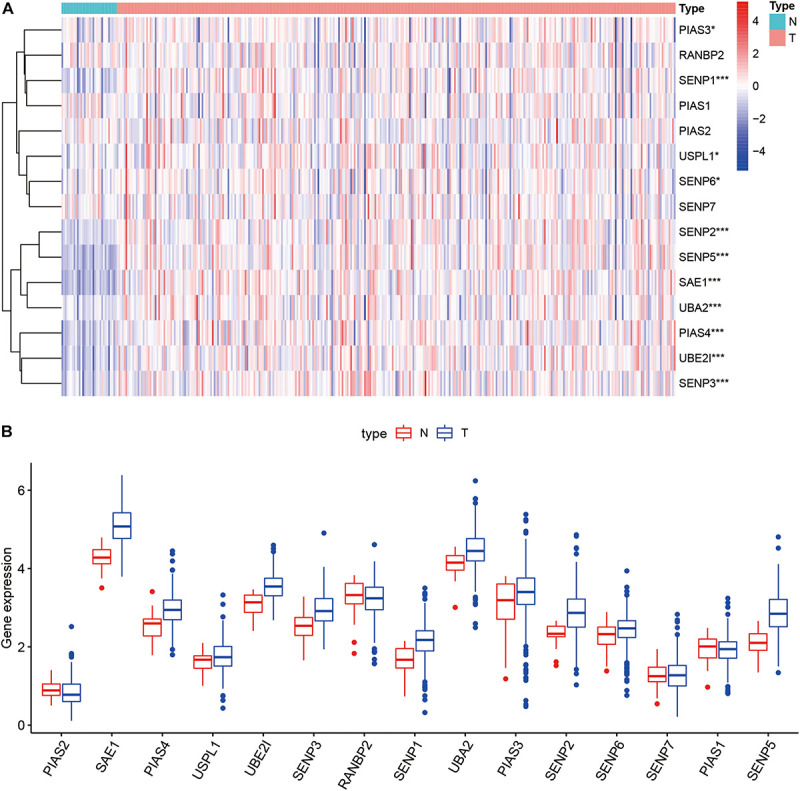
Heatmap and boxplot of differential expression of SUMOylation regulators. **(A)** The heatmap of SUMOylation regulators. **(B)** The boxplot of SUMOylation regulators.

**FIGURE 3:**
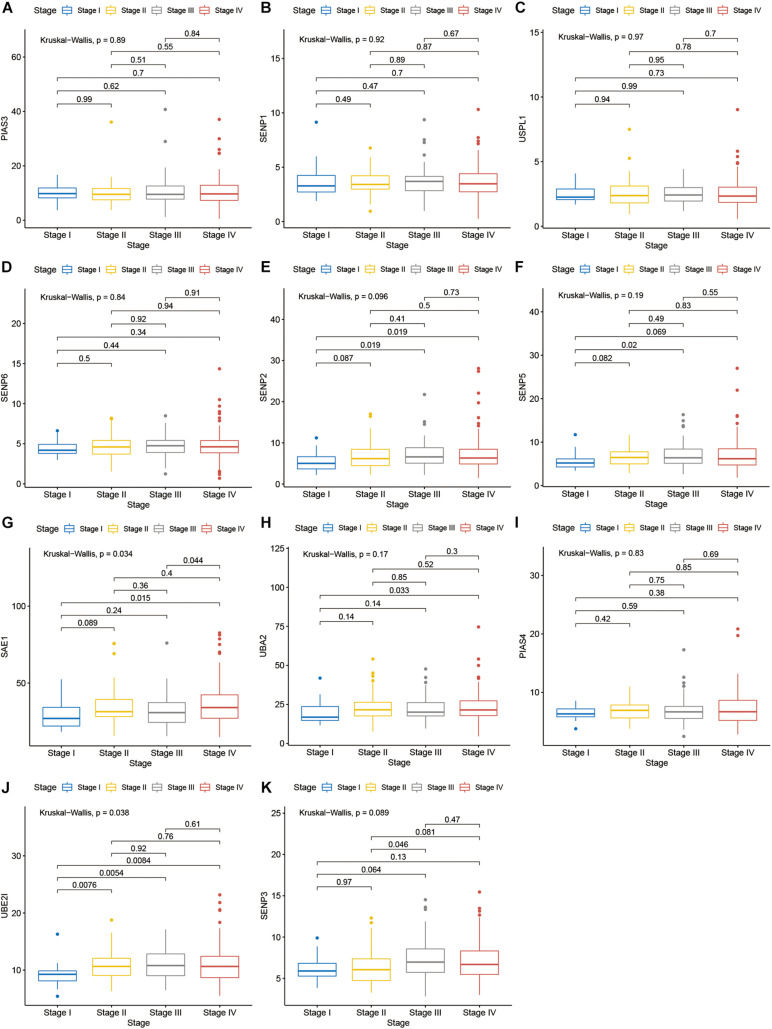
SAE1 and UBE2I had differential expressions with different stages. **(A)** PIAS3; **(B)** SENP1; **(C)** USPL1; **(D)** SENP6; **(E)** SENP2; **(F)** SENP5; **(G)** SAE1; **(H)** UBA2; **(I)** PIAS4; **(J)** UBE2I; **(K)** SENP3 in different stages.

**FIGURE 4:**
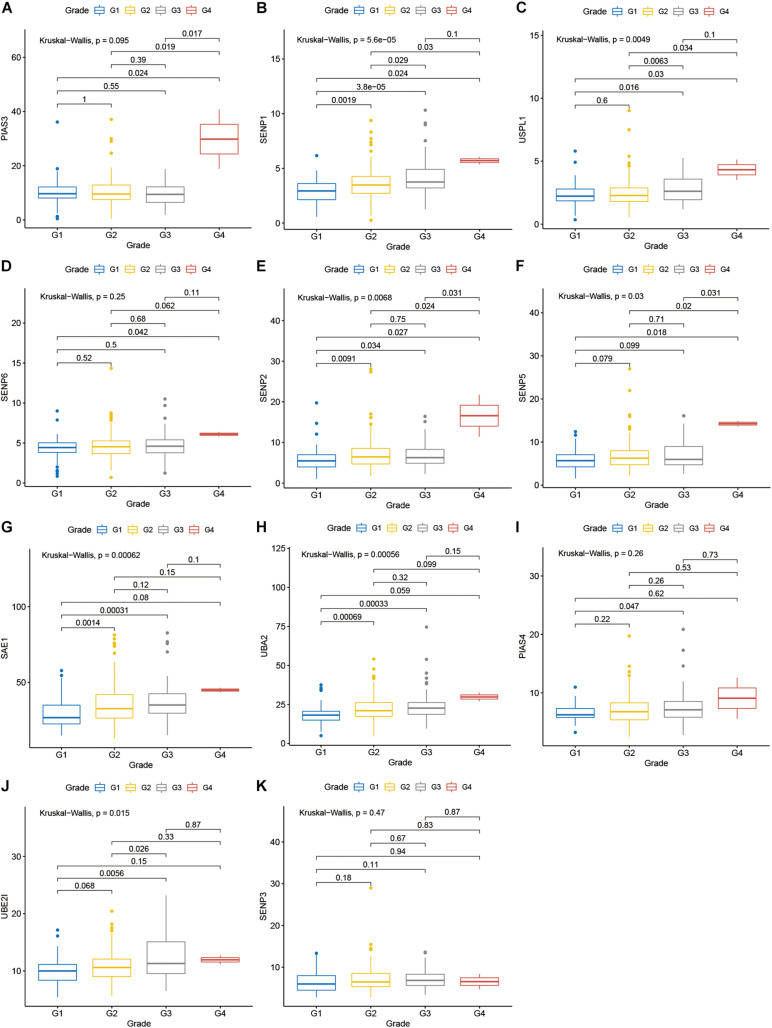
SENP1, USPL1, SENP2, SENP5, SAE1, UBA2, and UBE2I had differential expressions with different tumor grades. **(A)** PIAS3; **(B)** SENP1; **(C)** USPL1; **(D)** SENP6; **(E)** SENP2; **(F)** SENP5; **(G)** SAE1; **(H)** UBA2; **(I)** PIAS4; **(J)** UBE2I; **(K)** SENP3 in different grades.

Compared with normal tissues, 11 genes including PIAS3, RANBP2, USPL1, SENP6, SENP2, SENP5, SAE1, UBA2, PIAS4, UBE2I, and SENP3 were highly expressed in OSCC. These heatmap and histogram of these differentially expressed SUMOylation regulators are shown in Figures 2A,B. Among these 11 genes, SAE1 and UBE2I had differential expressions with different stages (Figures 3A–K). Moreover, SENP1, USPL1, SENP2, SENP5, SAE1, UBA2, and UBE2I had differential expressions with different tumor grades (Figures 4A–K). Subgroup analyses showed that the SUMOylation status might be various with different stages and grades. In the following analyses, this study was to analyze the prognosis of the overall OSCC patients regardless of different subgroups, which might be more generally applicable to this disease.

### GSEA

**FIGURE 5:**
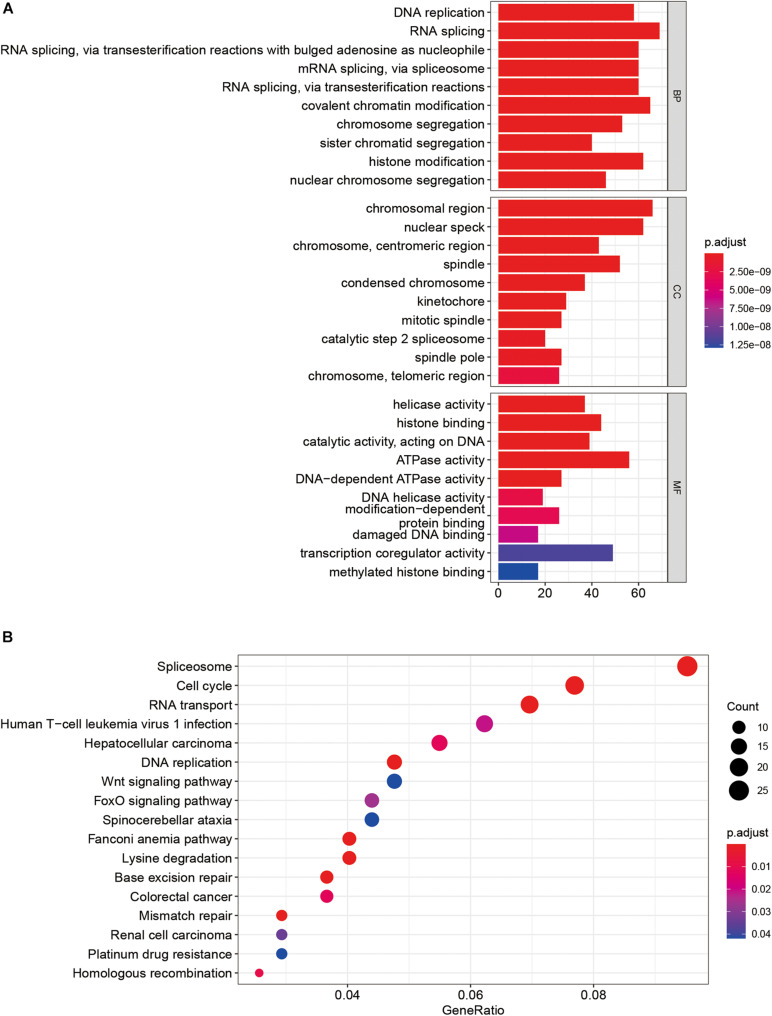
GSEA of SUMOylation regulators in OSCC. **(A)** GO analysis. **(B)** KEGG analysis.

GO enrichment analysis showed that these SUMOylation regulator-related genes might be related to DNA replication, RNA splicing, and histone binding pathways (Figure 5A). KEGG analysis enriched for these genes was linked to pathways such as Spliceosome, cell cycle, RNA transport, and DNA replication (Figure 5B).

### Prognosis Model of OSCC

**FIGURE 6:**
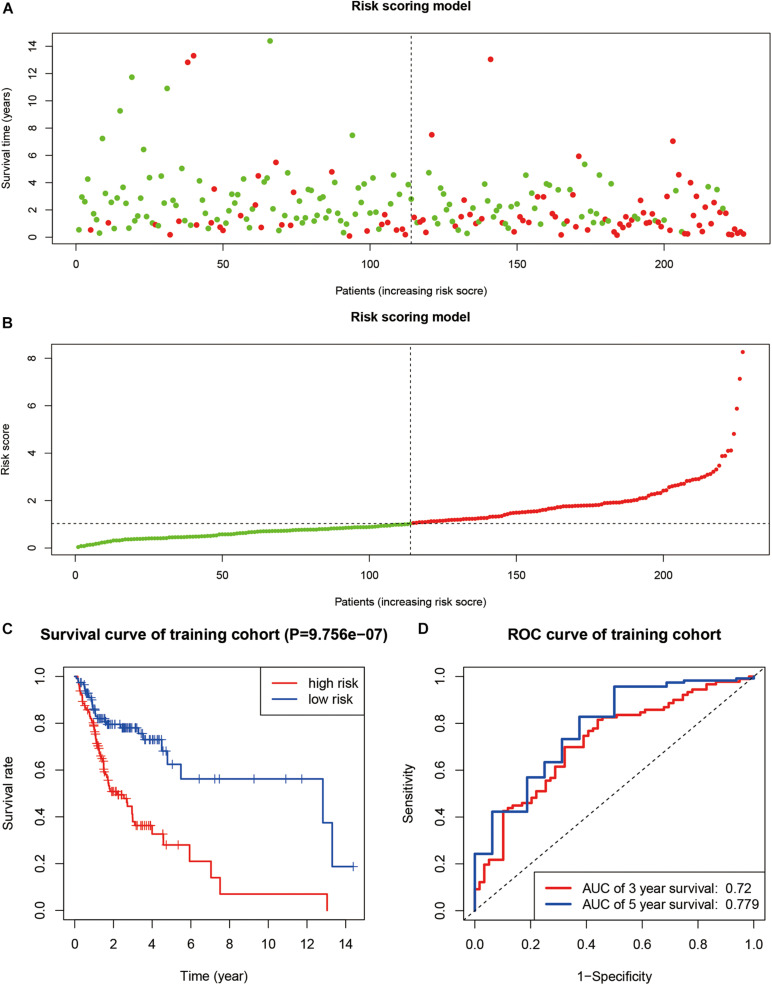
The construction of a prognosis model. **(A,B)** The survival status and risk score of each sample. **(C)** Kaplan–Meier curve in TCGA training group. **(D)** ROC curve in TCGA training group.

We identified a total of 683 SUMOylation regulator-related genes. Using univariate Cox regression in the TCGA training group, 37 genes were found to be associated with the overall survival of OSCC. Furthermore, a nine-gene prognostic model was established based on multivariate Cox regression. The risk score can be expressed as: Risk score = 0.123 × Expression_*TRIM37*_ + 0.062 × Expression_*UFM1*_ − 0.053 × Expression_*FUBP1*_ − 0.285 × Expression_*CCNT1*_ + 0.056 × Expression_*FXR1*_ + 0.305 × Expression_*HMG20A*_ − 0.291 × Expression_*RANBP3*_ + 0.457 × Expression_*SPATA5*_ + 0.126 × Expression_*DDX23*_. The survival status and risk score of each sample are shown in Figures 6A,B. The survival curve of the TCGA training group showed that cases with high-risk scores had worse overall survival status than cases with low-risk scores (*P* < 0.001, Figure 6C). The ROC curve showed that the 3-year and 5-year AUC were 0.720 and 0.779, respectively (Figure 6D).

**FIGURE 7:**
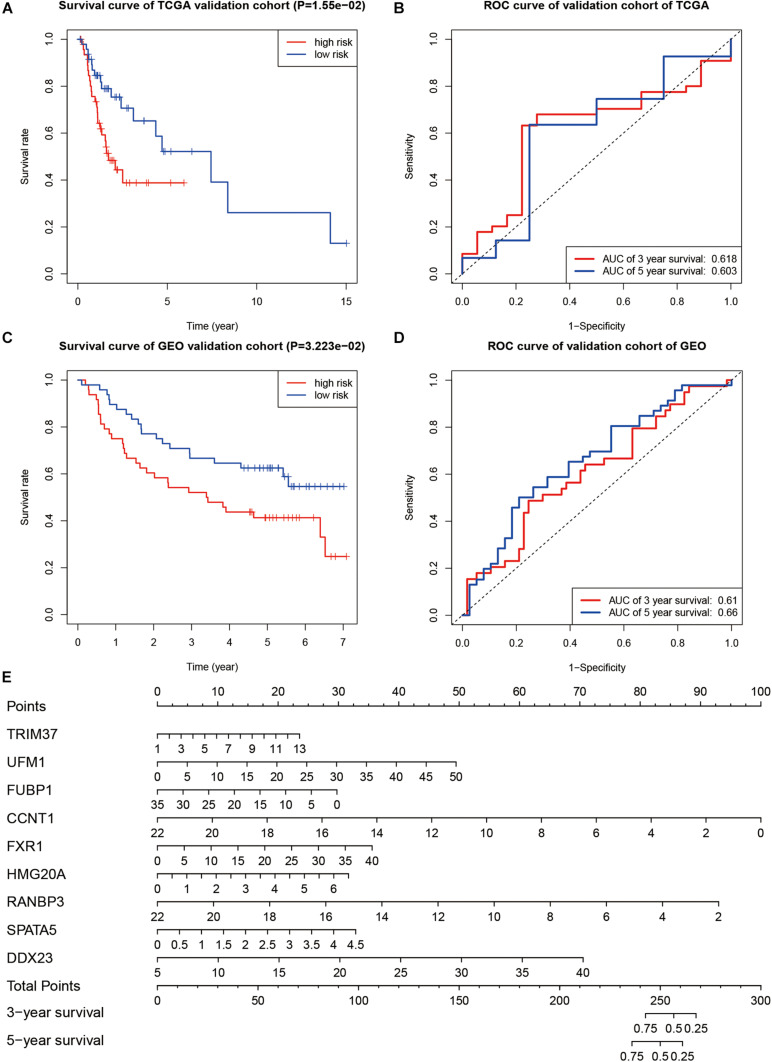
Evaluation of the prognostic ability. **(A)** Kaplan–Meier curve in TCGA validation group. **(B)** ROC curve in TCGA validation group. **(C)** Kaplan–Meier survival curve in the GEO validation group. **(D)** The ROC curve in the GEO validation group. **(E)** Nomogram to predict overall survival of OSCC.

In order to verify the prognostic ability of this model, we also drew the survival and ROC curves in the TCGA validation group and GEO validation group. In both TCGA validation group and GEO validation group, the overall survival of cases with high-risk scores were worse than that of cases with low-risk scores (*P* < 0.001, Figures 7A–C). The AUC values of the TCGA verification group were 0.618 (for 3 years) and 0.603 (for 5 years), respectively, as shown in Figure 7B. The AUC values of the GEO validation group were 0.610 (for 3 years) and 0.660 (for 5 years), respectively, as shown in Figure 7D. A nomogram was drawn according to the risk model as shown in Figure 7E.

## Discussion

Small ubiquitin-like modifier molecules in human beings include SUMO-1, SUMO-2, SUMO-3, and SUMO-4, and their sequences are commonly conserved (Yang et al., 2017). Through the tertiary catalysis of E1 activating enzyme, E2 conjugating enzyme, and E3 ligase, the SUMO molecules were efficiently bound to the lysine residues of substrates (Flotho and Melchior, 2013). Conversely, SUMO proteases can dissociate the SUMO molecules from the substrates instead of degrading the SUMO molecules (Kerscher et al., 2006). Most SUMOylation substrates exist in the nucleus. The substrates are in the rapid circulation of SUMO conjugation and deconjugation (Hay, 2005), but this dynamic process has an important regulatory effect on tumor cell proliferation and genome stability (Eifler and Vertegaal, 2015).

p53 is one famous tumor suppressor. Studies have shown that after p53 is covalently conjugated by SUMO-1, it cannot bind to DNA and promote p53-dependent transcription. The SUMOylation of p53 at the K386 prevents p53 acetylation induced by p300. However, p53 that has already been acetylated can still bind to DNA without being interfered by SUMOylation (Wu and Chiang, 2009). MDM2, an E3 ligase, can induce ubiquitination and degradation of p53 and transfer it from nucleus to cytoplasm (Carter et al., 2007). SKI can enhance the SUMOylation of MDM2, thereby upregulating the activity of MDM2 and reducing the level of p53 (Ding et al., 2012). In addition, β-catenin, the key protein of the WNT pathway, is associated with poor prognosis. In multiple myeloma, SUMOylation has been reported to inhibit ubiquitin–proteasomal mediated degradation of β-catenin, thereby upregulating the Wnt/β-catenin pathway and promoting cell proliferation (Huang et al., 2015). These studies show that studies of SUMOylation are of great significance for tumor pathogenesis and targeted therapy. However, current research on the molecular mechanism of SUMOylation in OSCC is quite limited.

By analyzing the differential expression of SUMOylation regulators in OSCC between tumor tissues and normal tissues, we found that the 11 genes including PIAS3, RANBP2, USPL1, SENP6, SENP2, SENP5, SAE1, UBA2, PIAS4, UBE2I, and SENP3 were highly expressed. Further subgroup analyses indicated that SAE1 and UBE2I had differential expressions with different stages, while SENP1, USPL1, SENP2, SENP5, SAE1, UBA2, and UBE2I had differential expressions with different tumor grades. The differential expressions of SUMOylation regulators indicated that SUMOylation might play an important role in the pathogenesis of OSCC. These differentially expressed SUMOylation regulators have been reported in some kinds of tumors. For instance, the expression of SAE1 increases with the level of glioma. SAE1 can increase the SUMOylation of Akt and promote the progression of glioma (Yang et al., 2019). UBE2I, the unique E2 conjugating enzyme, is highly expressed in osteosarcoma, and silencing UBE2I can induce the dissociation of Cx43 and SUMO-1, thus inhibiting the proliferation and migration of osteosarcoma cells (Zhang et al., 2018). In addition, UBA2 can promote the progression of colorectal cancer and is expected to become a potential therapeutic target for colorectal cancer (Cheng et al., 2018).

This study showed that SUMOylation regulator-related genes in OSCC were enriched in biological functions such as DNA replication, RNA splicing, cell cycle, RNA transport, and DNA replication. There are finite reports that SUMOylation regulators participate in mediating functions in OSCC. Research has shown that SENP3 is highly expressed in OSCC and may be related to cellular oxidative stress (Sun et al., 2013). SENP5 is mainly expressed in the nucleus, and compared with adjacent tissues, the expression in OSCC is increased (Ding et al., 2008). So far, the expression of more SUMOylation regulators and their involvement in OSCC pathogenesis are still unclear.

In this study, a prognostic model of OSCC was constructed based on nine SUMOylation regulator-related genes including TRIM37, UFM1, FUBP1, CCNT1, FXR1, HMG20A, RANBP3, SPATA5, and DDX23. The prognostic model for OSCC performed well in classifying patients with different overall survival. It might be useful for tumor prognosis evaluation and targeted therapy. Furthermore, valproic acid can reduce the expression levels of SUMO-1 and SUMO-2 in OSCC. It can increase the distribution of cell in G1 phase and reduced distribution of S phase, resulting in anti-tumor effects. However, the specific target of valproic acid needs to be further studied (Sang et al., 2016).

The main limitation of this study was that the main work was based on sequencing analyses. More basic *in vitro* and *in vivo* experiments to explore molecular mechanisms need to be further carried out.

## Conclusion

Compared with normal tissues, 11 genes including PIAS3, RANBP2, USPL1, SENP6, SENP2, SENP5, SAE1, UBA2, PIAS4, UBE2I, and SENP3 were highly expressed in OSCC. It is suggested that the SUMOylation regulator-related genes might be related to biological functions such as DNA replication, RNA splicing, cell cycle, RNA transport, and DNA replication. The prognostic model based on nine SUMOylation-regulated genes (TRIM37, UFM1, FUBP1, CCNT1, FXR1, HMG20A, RANBP3, SPATA5, and DDX23) had a strong ability to predict the prognosis of OSCC. This study might provide targets for prognostic evaluation and targeted therapy of patients with OSCC.

## Data Availability Statement

The original contributions presented in the study are included in the article/supplementary material, further inquiries can be directed to the corresponding author/s.

## Author Contributions

XL conducted data analysis and manuscript draft. YM conducted experiment design and final inspection. Both authors contributed to the article and approved the submitted version.

## Conflict of Interest

The authors declare that the research was conducted in the absence of any commercial or financial relationships that could be construed as a potential conflict of interest.

## Footnotes

1 https://cancergenome.nih.gov/

2 https://www.ncbi.nlm.nih.gov/geo/

## References

[B1] Carter S. Bischof O. Dejean A. Vousden K. H. (2007). C-terminal modifications regulate MDM2 dissociation and nuclear export of p53. *Nat. Cell Biol.* 9 428–435. 10.1038/ncb1562 17369817

[B2] Celen A. B. Sahin U. (2020). Sumoylation on its 25th anniversary: mechanisms, pathology, and emerging concepts. *FEBS J.* 287 3110–3140. 10.1111/febs.15319 32255256

[B3] Chang H. M. Yeh E. T. H. (2020). SUMO: from Bench to Bedside. *Physiol. Rev.* 100 1599–1619. 10.1152/physrev.00025.2019 32666886 PMC7717128

[B4] Cheng H. Sun X. Li J. He P. Liu W. Meng X. (2018). Knockdown of Uba2 inhibits colorectal cancer cell invasion and migration through downregulation of the Wnt/β-catenin signaling pathway. *J. Cell Biochem.* 119 6914–6925. 10.1002/jcb.26890 29744931

[B5] Chinn S. B. Myers J. N. (2015). Oral Cavity Carcinoma: current Management, Controversies, and Future Directions. *J. Clin. Oncol.* 33 3269–3276. 10.1200/jco.2015.61.2929 26351335 PMC5320919

[B6] Dhingra N. Zhao X. (2019). Intricate SUMO-based control of the homologous recombination machinery. *Genes Dev.* 33 1346–1354. 10.1101/gad.328534.119 31575678 PMC6771382

[B7] Ding B. Sun Y. Huang J. (2012). Overexpression of SKI oncoprotein leads to p53 degradation through regulation of MDM2 protein sumoylation. *J. Biol. Chem.* 287 14621–14630. 10.1074/jbc.m111.301523 22411991 PMC3340287

[B8] Ding X. Sun J. Wang L. Li G. Shen Y. Zhou X. (2008). Overexpression of SENP5 in oral squamous cell carcinoma and its association with differentiation. *Oncol. Rep.* 20 1041–1045. 18949399

[B9] Eifler K. Vertegaal A. C. O. (2015). SUMOylation-Mediated Regulation of Cell Cycle Progression and Cancer. *Trends Biochem. Sci.* 40 779–793. 10.1016/j.tibs.2015.09.006 26601932 PMC4874464

[B10] Flotho A. Melchior F. (2013). Sumoylation: a regulatory protein modification in health and disease. *Annu. Rev. Biochem.* 82 357–385. 10.1146/annurev-biochem-061909-093311 23746258

[B11] Harsha C. Banik K. Ang H. L. Girisa S. Vikkurthi R. Parama D. (2020). Targeting AKT/mTOR in Oral Cancer: mechanisms and Advances in Clinical Trials. *Int. J. Mol. Sci.* 21:3285. 10.3390/ijms21093285 32384682 PMC7246494

[B12] Hay R. T. (2005). SUMO: a history of modification. *Mol Cell* 18 1–12. 10.1016/j.molcel.2005.03.012 15808504

[B13] Huang H. J. Zhou L. L. Fu W. J. Zhang C. Y. Jiang H. Du J. (2015). β-catenin SUMOylation is involved in the dysregulated proliferation of myeloma cells. *Am. J. Cancer Res.* 5 309–320. 25628940 PMC4300696

[B14] Joseph A. W. D’Souza G. (2012). Epidemiology of human papillomavirus-related head and neck cancer. *Otolaryngol. Clin. North Am.* 45 739–764. 10.1016/j.otc.2012.04.003 22793850

[B15] Kerscher O. Felberbaum R. Hochstrasser M. (2006). Modification of proteins by ubiquitin and ubiquitin-like proteins. *Annu. Rev. Cell Dev. Biol.* 22 159–180. 10.1146/annurev.cellbio.22.010605.093503 16753028

[B16] Kroonen J. S. Vertegaal A. C. O. (2020). Targeting SUMO Signaling to Wrestle Cancer. *Trends Cancer* 7 496–510. 10.1016/j.trecan.2020.11.009 33353838

[B17] Moore S. R. Johnson N. W. Pierce A. M. Wilson D. F. (2000). The epidemiology of mouth cancer: a review of global incidence. *Oral Dis.* 6 65–74. 10.1111/j.1601-0825.2000.tb00104.x 10702782

[B18] Neville B. W. Day T. A. (2002). Oral cancer and precancerous lesions. *CA Cancer J. Clin.* 52 195–215. 10.3322/canjclin.52.4.195 12139232

[B19] Petersen P. E. (2003). The World Oral Health Report 2003: continuous improvement of oral health in the 21st century–the approach of the WHO Global Oral Health Programme. *Community Dent. Oral Epidemiol.* 31 3–23. 10.1046/j.2003.com122.x 15015736

[B20] Roy D. Sadanandom A. (2021). SUMO mediated regulation of transcription factors as a mechanism for transducing environmental cues into cellular signaling in plants. *Cell. Mol. Life Sci.* 78 2641–2664. 10.1007/s00018-020-03723-4 33452901 PMC8004507

[B21] Roy N. K. Monisha J. Padmavathi G. Lalhruaitluanga H. Kumar N. S. Singh A. K. (2019). Isoform-Specific Role of Akt in Oral Squamous Cell Carcinoma. *Biomolecules* 9:253. 10.3390/biom9070253 31252679 PMC6681224

[B22] Sang Z. Sun Y. Ruan H. Cheng Y. Ding X. Yu Y. (2016). Anticancer effects of valproic acid on oral squamous cell carcinoma via SUMOylation in vivo and in vitro. *Exp. Ther. Med.* 12 3979–3987. 10.3892/etm.2016.3907 28101176 PMC5228083

[B23] Sun Z. Hu S. Luo Q. Ye D. Hu D. Chen F. (2013). Overexpression of SENP3 in oral squamous cell carcinoma and its association with differentiation. *Oncol. Rep.* 29 1701–1706. 10.3892/or.2013.2318 23467634 PMC3658864

[B24] Wu S. Y. Chiang C. M. (2009). Crosstalk between sumoylation and acetylation regulates p53-dependent chromatin transcription and DNA binding. *EMBO J.* 28 1246–1259. 10.1038/emboj.2009.83 19339993 PMC2683057

[B25] Yang Y. He Y. Wang X. Liang Z. He G. Zhang P. (2017). Protein SUMOylation modification and its associations with disease. *Open Biol.* 7:170167. 10.1098/rsob.170167 29021212 PMC5666083

[B26] Yang Y. Liang Z. Xia Z. Wang X. Ma Y. Sheng Z. (2019). SAE1 promotes human glioma progression through activating AKT SUMOylation-mediated signaling pathways. *Cell Commun. Signal.* 17:82. 10.1186/s12964-019-0392-9 PMC6659289 31345225

[B27] Zhang D. Yu K. Yang Z. Li Y. Ma X. Bian X. (2018). Silencing Ubc9 expression suppresses osteosarcoma tumorigenesis and enhances chemosensitivity to HSV-TK/GCV by regulating connexin 43 SUMOylation. *Int. J. Oncol.* 53 1323–1331. 10.3892/ijo.2018.4448 29956745

